# The Role of Dual-Energy CT in Differentiating Adrenal Adenomas from Metastases: A Comprehensive Narrative Review

**DOI:** 10.3390/jpm15040131

**Published:** 2025-03-28

**Authors:** Francesco Tiralongo, Cristina Mosconi, Pietro Valerio Foti, Aldo Eugenio Calogero, Sandro La Vignera, Corrado Ini’, Davide Giuseppe Castiglione, Emanuele David, Stefania Tamburrini, Sebastiano Barbarino, Stefano Palmucci, Antonio Basile

**Affiliations:** 1 Radiology Unit 1, Department of Medical Surgical Sciences and Advanced Technologies “GF Ingrassia”, University Hospital Policlinico “G. Rodolico-San Marco”, University of Catania, 95123 Catania, Italy; pietrofoti@hotmail.com (P.V.F.); corrado.ini@gmail.com (C.I.); davidegiuseppecastiglione@gmail.com (D.G.C.); emanuele.david@unict.it (E.D.); sebbar98@gmail.com (S.B.); basile.antonello73@gmail.com (A.B.); 2 Department of Radiology, IRCCS Azienda Ospedaliero Universitaria Di Bologna, Via Albertoni 15, 40138 Bologna, Italy; cristina.mosconi@aosp.bo.it; 3 Department of Clinical and Experimental Medicine, University of Catania, 95123 Catania, Italy; aldo.calogero@unict.it (A.E.C.); sandrolavignera@unict.it (S.L.V.); 4 Department of Radiology, Ospedale del Mare, ASL NA1 Centro, 80147 Naples, Italy; tamburrinistefania@gmail.com; 5 UOSD IPTRA, Department of Medical Surgical Sciences and Advanced Technologies “GF Ingrassia”, University Hospital Policlinico “G. Rodolico-San Marco”, University of Catania, 95123 Catania, Italy; spalmucci@unict.it

**Keywords:** adrenocortical adenoma, neoplasm, adrenal gland, dual-energy computed tomography, virtual non-contrast imaging, fat fraction, spectral imaging

## Abstract

Dual-energy CT (DECT) has emerged as a novel imaging modality that offers a multiparametric approach for noninvasive adrenal lesion characterization. This narrative review examines recent advances in DECT—including virtual non-contrast imaging, iodine density quantification, spectral curve analysis, and material density mapping—for differentiating benign adrenal adenomas from metastases. Conventional CT techniques rely primarily on unenhanced attenuation measurements and contrast washout kinetics; however, these methods may be limited in evaluating lipid-poor adenomas, and in cases where imaging features overlap with metastatic lesions. Although virtual non-contrast imaging with DECT tends to overestimate attenuation relative to true non-contrast scans, the recalibration of diagnostic thresholds and integration with complementary parameters, such as the iodine density-to-virtual non-contrast attenuation ratio, can significantly enhance sensitivity and specificity. Additional parameters, including fat fraction analysis and the evaluation of attenuation changes across energy spectra, further refine tissue characterization by quantifying intracellular lipid content and vascularity. Material density analysis has demonstrated near-perfect diagnostic accuracy in select studies. By tailoring imaging evaluation to the unique spectral and compositional features of each adrenal lesion, DECT contributes to a more personalized diagnostic approach. This individualization allows for better differentiation between benign and malignant findings, potentially avoiding unnecessary interventions and enabling more targeted clinical management. Despite these promising developments, challenges remain regarding the standardization of acquisition protocols, optimization of diagnostic thresholds, and minimization of interobserver variability. Emerging radiomics and machine learning applications may further automate lesion classification and improve diagnostic accuracy. Thus, DECT holds considerable potential to improve diagnostic confidence, reduce radiation exposure, and streamline the management of patients with adrenal incidentalomas, although further multicenter validation is warranted.

## 1. Introduction

Adrenal lesions are frequently detected as incidental findings on cross-sectional imaging, with an estimated prevalence ranging from 3% to 7% in adults [1]. Although the majority of these lesions are benign adrenal adenomas—often characterized by intracellular lipid content—the differential diagnosis must also consider malignant processes such as adrenal metastases [2,3]. Accurate and noninvasive differentiation between adenomas and metastases is critical, as management strategies differ considerably. Conventional CT protocols have traditionally relied on the measurement of unenhanced attenuation values (typically using a threshold of 10 HU for lipid-rich adenomas) and contrast washout kinetics [4,5]. However, these approaches are sometimes limited, particularly in the evaluation of lipid-poor adenomas, and in cases where contrast-enhanced imaging yields overlapping features with metastases [4,5,6].

Recent advances in dual-energy CT (DECT) have allowed for the acquisition of datasets at two distinct photon energy levels, thereby enabling material differentiation based on unique attenuation profiles, providing new opportunities for tissue characterization [7,8].

This technology enables the generation of multiple postprocessed images and quantitative maps, including virtual non-contrast images, iodine density maps, spectral attenuation curves, and material density maps. These additional parameters can yield insights into iodine uptake, fat content, and effective atomic number, potentially improving the diagnostic accuracy of adrenal lesion characterization [8,9]. Numerous studies have evaluated various DECT parameters in adrenal imaging in recent years.

This review aims to integrate and critically discuss these findings, addressing the technical principles of DECT and evaluating how each parameter contributes to improved lesion characterization.

## 2. Technical Principles of Dual-Energy CT

Dual-energy CT systems acquire imaging data at two distinct energy levels, typically implemented via dual-source systems, rapid kilovoltage switching, or dual-layer detector configurations. This simultaneous acquisition of high- and low-energy data exploits the differential absorption of X-rays by various materials governed by their atomic composition [8]. The fundamental advantage of DECT is its ability to differentiate tissues based on subtle differences in energy-dependent attenuation, providing the foundation for generating multiple reconstructions that extend beyond conventional single-energy CT. Commonly derived images include virtual non-contrast images, iodine density maps, material density maps, and spectral attenuation curves that depict CT numbers as a function of photon energy [8]. These advanced reconstructions allow for the quantitative assessment of tissue properties such as iodine content, fat fraction, and effective atomic number, thereby facilitating a more refined characterization of adrenal lesions [10].

## 3. Virtual Non-Contrast (VNC) Imaging

Virtual non-contrast (VNC) imaging is one of the principal applications of DECT. By applying material decomposition algorithms to contrast-enhanced datasets, VNC imaging digitally subtracts iodine to simulate the appearance of a true non-contrast (TNC) scan.

The resulting images have the potential to reduce the radiation dose by obviating the need for an additional unenhanced scan.

This capability is particularly valuable in adrenal imaging, where the measurement of unenhanced attenuation is critical for identifying lipid-rich adenomas.

Ho et al., in a study involving 23 adrenal nodules, compared VNC attenuation values with TNC values on a dual-source CT scanner [11]. The authors found no statistically significant difference between the two sets of measurements across adenomas (both lipid-rich and lipid-poor) and metastases. However, a slight overestimation (mean difference of 1.8 HU) was observed, which was considered clinically acceptable [11].

Nagayama and colleagues evaluated VNC attenuation alongside iodine density in differentiating adrenal adenomas from metastases [12]. Their study demonstrated a strong positive correlation between the VNC and TNC values (r = 0.92). However, VNC images tended to overestimate lesion attenuation, particularly in adenomas (mean VNC attenuation of 21 HU vs. 10 HU on true non-contrast images). Consequently, the conventional 10-HU threshold applied to the TNC images yielded a sensitivity of only 11% when applied to the VNC images. Optimizing the threshold (e.g., raising the cutoff to 22–29 HU) improved sensitivity (up to 79%), but it still lagged behind the performance of TNC images (85% sensitivity) [12].

Cao and colleagues reported that VNC images generated from dual-layer dual-energy CT (dlDECT) systematically overestimated attenuation values in adrenal nodules. Using the conventional 10-HU threshold resulted in a sensitivity of only 20% for diagnosing lipid-rich adenomas [13]. By adjusting the threshold to ≤22 HU, the sensitivity improved to 82% with an acceptable specificity of 85% [13]. The authors postulated that the overestimation was related to the presence of iodine residuals or the potential limitations of the two-material decomposition algorithm in the context of lesions with complex composition.

Loonis et al. evaluated VNC-derived metrics on single-phase contrast-enhanced DECT and found that a VNC threshold of approximately 13 HU provided high diagnostic accuracy (AUC = 0.89) for distinguishing adenomas from metastases [14]. Their data reinforced the notion that VNC imaging could serve as a reliable surrogate for TNC imaging if appropriate threshold adjustments are made.

The literature consistently demonstrates that VNC imaging is a promising tool for adrenal lesion characterization. However, the systematic overestimation of attenuation values relative to true non-contrast imaging necessitates the adjustment of diagnostic thresholds. An optimal threshold in the 13–22 HU range appears to balance sensitivity and specificity [12,13,14]. Importantly, the slight overestimation may be mitigated by combining VNC imaging with other DECT-derived parameters, such as iodine density, to improve overall diagnostic accuracy.

## 4. Iodine Density Quantification

In addition to VNC imaging, DECT facilitates the quantitative assessment of iodine concentration within adrenal lesions during the contrast-enhanced phase.

Iodine density maps were generated by quantifying the amount of iodine within a lesion, measured in mg/mL, during the contrast-enhanced phase. This parameter reflects the degree of vascularity and contrast uptake, which may differ between adrenal adenomas and metastases.

Adenomas generally show rapid contrast enhancement followed by washout, whereas metastases often demonstrate more sustained enhancement patterns.

Using a dual-source CT system, Martin and colleagues evaluated the iodine density in adrenal lesions [15]. Their study revealed that in the portal phase, the iodine density of adenomas was significantly lower than that of metastases (1.3 ± 0.4 mg/mL vs. 3.2 ± 1.4 mg/mL, respectively; *p* < 0.001) [15]. The authors postulated that differences in the underlying histopathologic features of the metastases—particularly those arising from renal carcinoma, which may contain intrinsic lipid—could account for this discrepancy relative to other studies [15].

In contrast, Nagayama et al. reported that iodine density was significantly higher in adenomas (2.4 mg/mL ± 0.8) compared with metastases (1.7 mg/mL ± 0.5) during the portal venous phase (*p* < 0.001). However, the diagnostic performance of iodine density alone (AUC = 0.79) was inferior to that of unenhanced attenuation measurements (AUC = 0.96) [12]. The authors attributed this finding to the variability in contrast media kinetics and iodine uptake among the lesions.

Also, Wu et al. demonstrated that the iodine density of adenomas in the venous phase was higher than that of metastases (1.75 ± 0.93 mg/mL vs. 1.16 ± 0.55 mg/mL, *p* < 0.05), explaining how the difference between the Martin et al. results in that study was that the majority of metastases came from primary renal cell carcinoma [15,16].

In their series, adenomas (both lipid-rich and lipid-poor) showed distinct iodine density profiles compared with metastases, although absolute values varied according to the phase of imaging and the specific lesion characteristics [16].

Another study evaluated iodine density on a dual-source CT system during the portal venous phase; in particular, Winkelmann et al. reported no statistically significant difference in iodine density between adenomas and metastases (2.23 mg/mL vs. 1.44 mg/mL, respectively; *p* = 0.060) [17].

Iodine density is a key DECT parameter that provides valuable insights into lesion vascularity. The literature, however, presents conflicting results regarding absolute iodine density values between adenomas and metastases. Variability in contrast injection protocols, the timing of image acquisition (portal vs. delayed phases), scanner technology (dual-source vs. dual-layer detectors), and the predominant primary malignancy in metastases may contribute to these discrepancies. Consequently, while iodine density alone may not be sufficient for reliable differentiation, its integration into composite indices—such as the iodine density/VNC attenuation ratio—may enhance diagnostic performance.

## 5. Iodine Density-to-VNC Attenuation Ratio

Given that adenomas characteristically exhibit low non-contrast attenuation due to intracellular lipid and may simultaneously demonstrate significant iodine uptake during contrast-enhanced phases, the combination of these parameters into an iodine density-to-VNC attenuation ratio has been proposed as a robust metric for lesion characterization. This composite parameter leverages complementary information from both VNC imaging and iodine quantification to enhance sensitivity and specificity.

Nagayama et al. introduced the iodine density-to-VNC attenuation ratio, calculated as the iodine density divided by the VNC attenuation (multiplied by 100), as a promising parameter for differentiating adenomas from metastases [12]. Their study found that adenomas had a significantly higher mean iodine density-to-VNC ratio than metastases (15 ± 10 vs. 4.6 ± 1.4, respectively; *p* < 0.001). The combined parameter demonstrated an area under the receiver operating characteristic (ROC) curve (AUC) comparable to that of unenhanced attenuation (0.98 vs. 0.96) and achieved a sensitivity of 95% and specificity of 95% with optimized thresholds [12].

Wu et al. also evaluated the iodine-to-VNC ratio as part of a panel of spectral parameters [16]. Their analysis confirmed that this ratio had superior diagnostic performance in the venous phase compared with individual parameters, with a higher AUC and improved sensitivity and specificity relative to using either iodine density or VNC attenuation alone [16].

The iodine density-to-VNC attenuation ratio appears to be one of the most promising DECT parameters for differentiating adrenal adenomas from metastases. This ratio mitigates the limitations inherent in each measurement by integrating the complementary features of low VNC attenuation (reflecting intracellular lipid content) and high iodine uptake (reflecting vascularity).

## 6. Fat Fraction Analysis

Adrenal adenomas, particularly the lipid-rich subtype, contain significant amounts of intracellular fat. Fat fraction analysis using DECT is based on the differential attenuation characteristics of fat versus soft tissue at varying photon energy levels. Material decomposition techniques enable the quantification of fat content as a percentage, thereby providing an objective measure that may help distinguish adenomas from metastases, which generally lack significant fat content.

As reported by Winkelmann et al., adenomas exhibited a significantly higher median fat fraction (30.25% [IQR: 26.94, 34.84]) compared with metastases (16.32% [IQR: 7.65, 24.10]; *p* < 0.001). In their ROC analysis, a fat fraction threshold of ≤17.20% yielded an AUC of 0.86, with a sensitivity of 68.75% and a specificity of 93.75% [17].

Studies by Ju et al. and Martin et al. have similarly demonstrated that fat fraction analysis can differentiate between lipid-rich and lipid-poor adenomas and between adenomas and metastatic lesions [15,18]. Although absolute fat fraction thresholds vary among studies, the overall trend remains that adenomas consistently exhibit higher fat fractions than metastases.

Loonis et al. included the fat fraction as part of a multiparametric approach to single-phase contrast-enhanced DECT [14].

Fat fraction analysis directly assesses the intracellular lipid content, a hallmark of adrenal adenomas. The high specificity associated with fat fraction thresholds—often exceeding 90%—is particularly valuable in confirming the benign nature of a lesion. However, the sensitivity may be suboptimal, especially in cases of lipid-poor adenomas. Thus, fat fraction analysis is most effective when used in conjunction with other DECT parameters.

## 7. Mean Attenuation and Attenuation Changes

The mean attenuation values measured in Hounsfield units (HU) have long been the cornerstone of CT-based adrenal lesion characterization. Conventional non-contrast CT employs a threshold of 10 HU to differentiate lipid-rich adenomas from other lesions. However, dual-energy CT allows for the evaluation of attenuation at multiple energy levels (e.g., 80 vs. 140 kVp) and the assessment of attenuation changes across these energy levels. The magnitude of the attenuation change can provide insights into the lesion’s composition, as the presence of intracellular lipid tends to result in minimal variation between energy levels compared with lesions lacking fat.

Gupta and colleagues compared the mean attenuation values at 140 kVp and 80 kVp in adrenal lesions [19]. They found that adenomas, particularly lipid-rich ones, exhibited significantly lower mean attenuation values than metastatic lesions. In their study, the mean attenuation change between 140 kVp and 80 kVp was significantly lower in adenomas (0.4 ± 7.1 HU) compared with metastases (9.2 ± 4.3 HU; *p* < 0.003) [19]. These findings suggest that adenomas demonstrate less variability in attenuation across different energy levels due to their intrinsic lipid content.

Ju et al. assessed the spectral behavior of adrenal lesions using single-source dual-energy CT [18]. They observed that adenomas tended to show either an ascending or minimal change in attenuation as the photon energy increased, whereas metastases typically exhibited a descending spectral curve. The mean attenuation changes were statistically significant between the two lesion types, particularly the difference between 140 kVp and lower energy levels (e.g., 80 kVp or 40 keV) [18].

Other studies have corroborated that mean attenuation values and their changes across energy spectra are valuable parameters [15,19,20]. Martin et al. reported that the mean attenuation changes correlated with the degree of lipid content, and that thresholds based on these changes could effectively differentiate adenomas from metastases with high specificity [15]. Similarly, Mileto et al. demonstrated that evaluating the absolute attenuation differences between energy levels provided diagnostic information beyond that available from a single-energy acquisition [20].

These authors have further refined the analysis of attenuation changes by exploring the correlation between the mean attenuation at a given energy level and the corresponding change when compared to another energy level [21,22,23]. Their findings indicate that specific thresholds (e.g., a difference of >2–3 HU between 140 kVp and lower energies) can be used as reliable indicators for the diagnosis of adenomas. In particular, Shi et al. reported that mean attenuation changes (MAVC 80–140 kVp and MAVC 40–100 keV) yielded AUC values of 0.964, with sensitivities and specificities approaching 79% and 100%, respectively, when optimal cutoffs were applied [23].

The evaluation of mean attenuation and its changes across different energy levels provides a quantitative approach to adrenal lesion characterization that complements traditional unenhanced CT measurements. The ability to measure attenuation differences in a multiphasic manner is particularly useful in cases where the conventional 10 HU threshold may not be applicable, such as in lipid-poor adenomas. Although technical factors such as scanner type and acquisition parameters can influence absolute attenuation values, the relative change between energy levels appears to be a robust indicator of tissue composition.

## 8. Spectral Curve Analysis

Spectral curve analysis involves plotting the CT number (in HU) as a function of the photon energy (typically ranging from 40 to 140 keV) to generate a spectral attenuation curve. The shape and slope of this curve (s-SHC) provide insights into the effective atomic number and material composition of a lesion. Adenomas and metastases tend to exhibit distinct spectral curve patterns, with adenomas often showing an ascending or flat curve, and metastases exhibiting a descending curve.

In their study, Ju et al. demonstrated that the spectral curves of adrenal lesions differ significantly between adenomas and metastases [18]. For metastases, the CT number was highest at low photon energy (e.g., 40 keV) and decreased with increasing energy, yielding a descending curve. In contrast, adenomas showed lower CT numbers at low energy, with a tendency to increase or remain flat as the energy increased. The authors quantified the slope of the spectral curve (K) and found that the absolute value of the slope was significantly different between the two groups. A threshold based on the slope provided high sensitivity and specificity for differentiation [18].

Wu and colleagues also evaluated the spectral curve parameters, including the slope (s-SHC) and the effective atomic number (Z_eff) [16]. Their results indicated that spectral curve analysis was particularly useful in the venous phase, where differences in the slope between adenomas and metastases were most pronounced. The authors reported that combining spectral curve metrics with other DECT parameters further enhanced diagnostic accuracy.

Spectral curve analysis represents a sophisticated approach to lesion characterization that leverages the full potential of dual-energy imaging. The differential behavior of adenomas and metastases across the energy spectrum can be quantified by calculating the slope of the spectral curve and assessing the effective atomic number. Although spectral curve analysis requires dedicated software and expertise in interpretation, its potential to provide incremental diagnostic value is significant.

## 9. Material Density Analysis

Material density analysis involves the use of material decomposition algorithms to generate maps that quantify the relative densities of specific materials—such as fat, iodine, and water—within a lesion. By selecting appropriate material basis pairs (e.g., fat–iodine, iodine–fat, fat–water, water–fat), DECT can provide a detailed assessment of the tissue composition. This approach is especially useful in adrenal imaging, where the differentiation between lipid-rich adenomas and nonadenomas (including metastases) hinges on subtle differences in fat content and contrast uptake.

Mileto and colleagues employed dual-energy multidetector CT with material decomposition to assess adrenal lesions [20]. Their study demonstrated significant differences in material density values between adenomas and nonadenomas across multiple basis pairs. For instance, adenomas showed lower density values in fat-iodine images than metastases. Importantly, by using cross-validated optimal thresholds, the authors achieved a diagnostic sensitivity of 96% and a specificity of 100% for differentiating adenomas from nonadenomas, outperforming conventional unenhanced CT [20].

Morgan et al. focused on single-source rapid kilovolt-switching DECT and quantified material densities in units of mg/mL, indicating that fat density values derived from fat–iodine or fat–water basis pairs could effectively differentiate high-lipid-content adenomas from low-lipid-content lesions [21]. The strong correlations observed among different material density measurements (|r| > 0.95) supported the robustness of this technique.

The utility of material density maps was recently validated by establishing the optimal cutoff values (1009.5 mg/cm^3^) based on the water–iodine pair in the portal phase, achieving an AUC of 0.926 with both sensitivity and specificity around 92% [22].

By quantifying the relative proportions of fat, iodine, and water within adrenal lesions, material density analysis via DECT provides a more nuanced understanding of the lesion composition than conventional attenuation measurements alone. The high diagnostic performance reported in multiple studies suggests that material density analysis can effectively distinguish lipid-rich adenomas from metastases.

## 10. Discussion

The current literature clearly demonstrates that dual-energy CT offers a multiparametric framework for the noninvasive characterization of adrenal lesions. Despite its systematic overestimation of attenuation values, virtual non-contrast imaging remains a valuable surrogate for true non-contrast imaging when appropriate thresholds are applied. Several studies consistently show that VNC values correlate well with TNC values, although the overestimation requires the recalibration of diagnostic cutoffs (Table 1) [11,12,13]. When integrated with iodine density quantification to form the iodine density-to-VNC attenuation ratio, the diagnostic performance is dramatically enhanced, with studies reporting sensitivity and specificity rates approaching 95% and an AUC near 0.98.

Furthermore, DECT’s capability to assess spectral parameters provides additional discriminatory power. The evaluation of the spectral Hounsfield unit curve, effective atomic number, and normalized iodine density allows for a more nuanced analysis of lesion composition, particularly in lipid-poor adenomas where conventional metrics may be insufficient. Low-keV virtual monoenergetic imaging further accentuates the differences between adenomas and metastases, with the best differentiation often achieved at 40 keV [18].

The analysis of mean attenuation values across different energy levels and the corresponding attenuation changes (ΔHU) constitute another critical aspect of DECT.

Adenomas typically exhibit minimal changes in attenuation between high-energy and low-energy images, whereas metastases demonstrate a marked increase [19,23]. These quantitative differences in attenuation change provide direct evidence of the underlying tissue composition—specifically, the presence of intracellular lipid—and offer robust criteria for lesion differentiation.

Material density analysis, which enables the quantitative mapping of fat, iodine, and water within a lesion, represents perhaps the most precise DECT-derived parameter.

This technique can achieve near-perfect diagnostic accuracy, with sensitivities as high as 96% and specificity of 100% [20,21]. Additionally, single-source DECT systems, such as those employing rapid kilovolt-switching, offer the advantage of acquiring all necessary spectral data in a single scan, thereby reducing radiation exposure by eliminating the need for separate unenhanced acquisition.

Despite these promising findings, several technical considerations must be addressed before DECT can be widely adopted in clinical practice. Variability in scanner technology, acquisition protocols, contrast injection rates, and reconstruction algorithms can all influence the measured parameters. Moreover, interobserver variability and lesion heterogeneity further complicate the establishment of universally accepted diagnostic thresholds. Large-scale multicenter studies are therefore needed to standardize protocols and validate the optimal cutoff values for each DECT parameter.

From a clinical perspective, integrating these DECT-derived metrics into a unified diagnostic algorithm can potentially streamline patient management. The combination of VNC imaging, iodine density quantification, spectral analysis, and material density mapping may reduce the need for additional imaging studies—such as MRI, PET/CT, or delayed-phase CT—thereby lowering radiation exposure and healthcare costs while improving diagnostic confidence. In particular, the iodine density-to-VNC attenuation ratio is a promising marker that could serve as the cornerstone of DECT-based adrenal lesion evaluation.

Future research should focus on several key areas to fully realize the potential of DECT in adrenal imaging. Standardization of acquisition protocols—such as contrast injection rates, scan timing (arterial, venous, or delayed phases), and reconstruction algorithms—is essential to ensure the consistency of measurements.

Additionally, further investigation into the integration of radiomics and machine learning with DECT data is warranted [7]. Advanced computational techniques have the potential to combine multiple DECT-derived parameters into predictive models that could yield real-time, automated lesion classification. Such models may further improve diagnostic accuracy and reduce interobserver variability.

## 11. Conclusions

Dual-energy CT (DECT) improves noninvasive adrenal lesion characterization by combining virtual non-contrast imaging, iodine density measurement, spectral analysis, and material density mapping. This multiparametric approach differentiates benign adenomas from metastatic lesions with high sensitivity and specificity—even in lipid-poor cases—by recalibrating diagnostic thresholds and incorporating complementary parameters like the iodine density-to-VNC ratio. Additionally, evaluating mean attenuation changes and material density offers direct insights into tissue composition, with some studies reporting high accuracy. By enabling a tailored imaging workup based on individual lesion characteristics, DECT supports a more personalized approach to adrenal incidentaloma evaluation. It minimizes unnecessary follow-up or invasive procedures, aligning diagnostic pathways with each patient’s unique imaging phenotype. Although DECT promises enhanced diagnostic confidence and reduced radiation exposure, challenges such as protocol standardization and interobserver variability remain. Future advancements integrating radiomics and machine learning may further automate lesion classification and improve outcomes in the evaluation of adrenal incidentalomas.

## Figures and Tables

**Table 1:** Main characteristics of the selected studies.

Author, Year	Aim of the Study	Dual-Energy Technology	Main Results							
**Cano Alonso et al., 2023 [22]**	To assess the ability of dual-energy CT (DECT) monochromatic images and material decomposition maps to differentiate adrenal adenomas from metastases and propose optimal cutoff values.	Rapid-kilovoltage-switching DECT (GE Revolution CT, GE HealthCare, Waukesha, Wisconsin, United States) with material decomposition analysis (water–iodine and fat–water basis pairs) and monochromatic imaging (55, 70, and 140 keV).	**Phase and Parameter**	**AUC**	**Optimal Threshold**		**Sensitivity (%)**		**Specificity (%)**	
			**Arterial Phase (70 keV, HU)**	0.76	≤42.4		92		60	
			**Arterial Phase (140 keV, HU)**	0.94	≤18.9		88		94	
			**Portal Phase (55 keV, HU)**	0.76	≤95.4		68		84	
			**Portal Phase (70 keV, HU)**	0.82	≤58.4		80		84	
			**Portal Phase (140 keV, HU)**	0.90	≤16.35		96		84	
			**Diagnostic Performance of DECT Material Density Maps for Differentiating Adenomas and Metastases**							
			**Phase and Parameter**	**AUC**	**Optimal Threshold**		**Sensitivity (%)**		**Specificity (%)**	
			**Arterial Phase (Water–Iodine, mg/cm^3^)**	0.97	≥1012.5		88		96	
			**Portal Phase (Water–Iodine, mg/cm^3^)**	0.93	≥1009.5		92		92	
**Cao et al., 2021 [13]**	To evaluate the diagnostic performance of dual-layer dual-energy CT (dlDECT) in characterizing adrenal nodules and to determine whether virtual unenhanced (VUE) images can replace true unenhanced (TUE) acquisitions.	Dual-layer dual-energy CT (Philips iQon, Philips Medical Systems, Cleveland, Ohio, United States) with material decomposition-based virtual unenhanced imaging.	**Nodule Type**	**True Unenhanced Attenuation (Mean ± SD, HU)**			**Virtual Unenhanced Attenuation (Mean ± SD, HU)**			***p*-Value**
			**All Adrenal Nodules (*n* = 73)**	7.1 ± 19.8			20.0 ± 17.2			<0.05
			**Adenomas (*n* = 65)**	6.9 ± 12.8			20.5 ± 9.9			<0.0001
			**Virtual Unenhanced Attenuation Thresholds for Lipid-Rich Adenomas**				**Sensitivity (%)**		**Specificity (%)**	
			**≤10 HU (Standard Threshold)**				20 (9/45)		100 (26/26)	
			**≤22 HU (Adjusted Threshold)**				82 (37/45)		85 (22/26)	
**Gupta et al., 2010 [19]**	To evaluate whether dual-energy CT (DECT) can improve the diagnostic performance of CT in differentiating adrenal adenomas from metastatic lesions.	Single-source 64-MDCT scanner (GE LightSpeed VCT, GE HealthCare, Waukesha, Wisconsin, United States) with dual-energy software (Volume Dual Energy, GE Healthcare. Waukesha, Wisconsin, United States) using 140 kVp and 80 kVp acquisitions.	**Parameter**			**Adenomas (n = 26)**		**Metastases (n = 5)**		***p*-Value**
			**Mean Attenuation at 140 kVp (HU)**			2.2 ± 9.9		28.5 ± 2.9		<0.003
			**Mean Attenuation at 80 kVp (HU)**			2.6 ± 13.3		37.7 ± 6.0		<0.003
			**Mean Attenuation Change (80–140 kVp) (HU)**			0.4 ± 7.1		9.2 ± 4.3		<0.003
			**Mean Transverse Diameter (mm)**			15.9 ± 6.8		40.7 ± 21.9		-
			**Subgroup Analysis: Lipid-Rich vs. Lipid-Poor Adenomas vs. Metastases**							
			**Parameter**	**Lipid-Rich Adenomas (n = 20)**		**Lipid-Poor Adenomas (n = 6)**		**Metastases (n = 5)**		***p*-Value**
			**Mean Attenuation at 140 kVp (HU)**	−2.2 ± 6.0		16.6 ± 5.4		28.5 ± 2.9		<0.01
			**Mean Attenuation at 80 kVp (HU)**	−2.0 ± 10.7		17.9 ± 9.4		37.7 ± 6.0		<0.01
			**Mean Attenuation Change (80–140 kVp) (HU)**	0.2 ± 7.7		1.3 ± 4.6		9.2 ± 4.3		<0.01
**Ho et al., 2012 [11]**	To determine whether virtual unenhanced (VUE) attenuation values derived from contrast-enhanced dual-energy CT can reliably replace true unenhanced attenuation values in the characterization of adrenal nodules.	Dual-source CT (Siemens Somatom Definition, Siemens Healthineers, Forchheim, Germany) with 80 kVp and 140 kVp energy levels, using an iodine subtraction algorithm to generate virtual unenhanced images.	**Nodule Type**	**True Unenhanced Attenuation (Mean ± SD, HU)**			**Virtual Unenhanced Attenuation (Mean ± SD, HU)**			***p*-Value**
			**All Nodules (n = 23)**	12.9 ± 13.4			14.7 ± 15.1			0.20
			**Adenomas (n = 19)**	8.9 ± 10.4			10.3 ± 13.1			0.45
			**Lipid-Rich Adenomas (n = 9)**	−0.41 ± 2.6			2.8 ± 11.5			0.34
			**Lipid-Poor Adenomas (n = 10)**	19.6 ± 6.5			20.0 ± 9.6			0.83
			**Metastases (n = 4)**	32.9 ± 5.9			37.2 ± 7.0			0.01
**Ju et al., 2015 [18]**	To evaluate the diagnostic performance of nonenhanced single-source dual-energy CT (ssDECT) in differentiating adrenal metastases from adenomas.	Single-source dual-energy CT (GE Discovery CT750 HD, GE HealthCare, Waukesha, Wisconsin, United States) with fast tube voltage switching (80 and 140 kVp) and material decomposition analysis.	**Parameter**	**Metastases (n = 63, Median ± Range)**			**Adenomas (n = 64, Median ± Range)**			***p*-Value**
			**CT Number at 40 keV (HU)**	50.47 ± 29.93			-0.76 ± 33.04			<0.001
			**CT Number at 140 keV (HU)**	29.00 ± 9.36			13.73 ± 18.96			<0.001
			**Effective Atomic Number (Z-eff)**	7.76 ± 0.23			7.42 ± 0.32			<0.001
			**Fat Concentration (mg/mL)**	−177.37 ± 296.38			126.73 ± 328.07			<0.001
			**Spectral Curve Patterns in Adenomas and Metastases**							
			**Curve Type**	**Metastases (n = 63)**			**Adenomas (n = 64)**			***p*-Value**
			**Ascending (K > 0.1)**	3.2% (2/63)			60.9% (39/64)			<0.05
			**Straight (−0.1 ≤ K ≤ 0.1)**	20.6% (13/63)			21.9% (14/64)			-
			**Descending (K < −0.1)**	76.2% (48/63)			17.2% (11/64)			<0.05
			**Diagnostic Accuracy of ssDECT Parameters in Differentiating Adenomas and Metastases**							
			**Parameter**		**Threshold**	**Sensitivity (%)**		**Specificity (%)**		**AUC**
			**CT Number at 40 keV (HU)**		≥21.78	92.1		76.6		0.90
			**Effective Atomic Number (Z-eff)**		≥7.59	81.0		75.0		0.88
			**Fat Concentration (mg/mL)**		≤−73.98	82.8		73.0		0.84
**Loonis et al., 2023 [14]**	To compare the diagnostic performance of various dual-energy CT (DECT)-derived metrics, including virtual non-contrast (VNC) attenuation, fat fraction, iodine density, and relative enhancement ratio, for characterizing adrenal masses.	Dual-source DECT (Siemens SOMATOM Definition Flash and SOMATOM Force, Siemens Healthineers, Forchheim, Germany) using three-material decomposition (fat, iodine, soft tissue) and virtual non-contrast imaging.	**Parameter**	**Adenomas (Mean ± SD)**	**Nonadenomas (Mean ± SD)**	***p*-Value**	**AUC**	**Optimal Threshold**	**Sensitivity (%)**	**Specificity (%)**
			**Virtual Non-contrast (VNC) Attenuation (HU)**	18.5 ± 12.9	34.1 ± 8.9	<0.001	0.85	≤15.2 HU	39%	100%
			**Fat Fraction (%)**	24.3 ± 8.2	14.2 ± 5.6	<0.001	0.85	≥23.8%	59%	100%
			**Normalized Iodine Density**	0.34 ± 0.15	0.17 ± 0.17	<0.001	0.81	≥0.90	1%	100%
			**Relative Enhancement Ratio**	186% ± 96%	58% ± 59%	<0.001	0.87	≥214%	37%	100%
**Martin et al., 2018 [15]**	To evaluate the diagnostic performance of third-generation dual-source dual-energy CT (DECT) iodine and fat quantification in differentiating adrenal adenomas from metastases.	Third-generation dual-source DECT (Siemens SOMATOM Force, Siemens Healthineers, Forchheim, Germany) using material decomposition analysis for iodine density and fat fraction quantification.	**Parameter**	**Adenomas (Mean ± SD)**	**Metastases (Mean ± SD)**	***p*-Value**	**AUC**	**Optimal Threshold**	**Sensitivity (%)**	**Specificity (%)**
			**Unenhanced Attenuation (HU)**	7.2 ± 14.8	26.9 ± 16.2	<0.001	0.75	≥10.0 HU	64.9	73.1
			**Contrast-Enhanced Attenuation (HU)**	42.1 ± 16.0	90.6 ± 19.4	<0.001	0.80	≥46.5 HU	58.3	84.6
			**Iodine Density (mg/mL)**	1.3 ± 0.4	3.2 ± 1.4	<0.001	0.97	≥1.6 mg/mL	97.2	96.2
			**Fat Fraction (%)**	34.2 ± 12.6	10.7 ± 7.8	<0.001	0.93	≤17.7%	89.2	88.5
**Mileto et al., 2015 [20]**	To determine whether contrast-enhanced dual-energy multidetector CT (DECT) with material decomposition analysis can differentiate adrenal adenomas from nonadenoma and compare its performance with that of non-enhanced CT.	Dual-energy multidetector CT (GE Discovery CT750 HD, GE HealthCare, Waukesha, Wisconsin, United States) with fast tube voltage switching (80 and 140 kVp) and material decomposition (fat–iodine and fat–water basis pairs).	**Parameter**	**Adenomas (Mean ± SD)**	**Nonadenomas (Mean ± SD)**	***p*-Value**	**AUC**	**Optimal Threshold**	**Sensitivity (%)**	**Specificity (%)**
			**Unenhanced Attenuation (HU)**	5.5 ± 18.2	32.3 ± 8.8	<0.0001	-	≤10.0 HU	67	100
			**Fat–Iodine Density (mg/cm^3^)**	970.4 ± 17.2	1012.3 ± 9.3	<0.0001	-	≤997 mg/cm^3^	96	100
			**Iodine–Fat Density (mg/cm^3^)**	2.5 ± 0.3	4.5 ± 1.5	<0.0001	-	≤3.0 mg/cm^3^	96	100
			**Fat–Water Density (mg/cm^3^)**	−666.7 ± 154.8	−2141.8 ± 953.2	<0.0001	-	≥−950 mg/cm^3^	96	100
			**Water–Fat Density (mg/cm^3^)**	1628.4 ± 177.3	3225.0 ± 986.1	<0.0001	-	≤1963.6 mg/cm^3^	96	100
**Morgan et al., 2013 [21]**	To evaluate whether single-source rapid kilovolt (peak)-switching dual-energy multidetector CT (RSDECT) can differentiate high-lipid-content (HLC) from low-lipid-content (LLC) adrenal lesions.	Single-source rapid kilovolt (peak)-switching DECT (GE Discovery CT750 HD, GE HealthCare, Waukesha, Wisconsin, United States) with material decomposition imaging using water–iodine and fat–iodine basis pairs.	**Parameter**	**HLC Adenomas (Mean ± SD)**		**LLC Adenomas (Mean ± SD)**		**Metastases (Mean ± SD)**		***p*-Value**
			**Unenhanced Attenuation (HU)**	−8.5 ± 13.9		27.4 ± 8.8		40.9 ± 12.3		<0.001
			**Attenuation at 140 keV (HU)**	5.44 ± 8.8		24.0 ± 4.4		28.5 ± 13.7		<0.001
			**Fat-Iodine Density (mg/mL)**	986 ± 8		1002 ± 4		1006 ± 13		<0.001
			**Water-Iodine Density (mg/mL)**	994 ± 8		1011 ± 5		1014 ± 13		<0.001
			**ROC Analysis for Differentiating HLC and LLC Lesions**							
			**RSDECT Parameter**	**AUC**	**Optimal Threshold**		**Sensitivity (%)**		**Specificity (%)**	
			**140 keV Attenuation (HU)**	0.929	≤9.5 HU		64%		94.4%	
			**Fat-Iodine Density (mg/mL)**	0.917	≤987 mg/mL		59%		94.4%	
			**Water-Iodine Density (mg/mL)**	0.912	≤994 mg/mL		50%		94.4%	
**Nagayama et al., 2020 [12]**	To determine whether virtual non-contrast (VNC) attenuation, iodine density, and their combination enable reliable differentiation between adrenal adenomas and metastases using portal venous phase dual-energy CT.	Dual-layer spectral detector CT (Philips iQon Spectral CT, Philips Medical Systems, Cleveland, Ohio, United States).	**Parameter**	**Adenomas (Mean ± SD)**	**Metastases (Mean ± SD)**	***p*-Value**	**AUC**	**Optimal Threshold**	**Sensitivity (%)**	**Specificity (%)**
			**Unenhanced Attenuation (HU)**	10 ± 13	35 ± 6	<0.001	0.96	≤22 HU	85	96
			**Virtual Non-contrast (VNC) Attenuation (HU)**	21 ± 10	36 ± 6	<0.001	0.91	≤29 HU	79	95
			**Iodine Density (mg/mL)**	2.4 ± 0.8	1.7 ± 1.0	<0.001	0.79	≥1.82 mg/mL	78	71
			**Iodine/VNC Ratio**	14.5 ± 10.2	4.6 ± 1.4	<0.001	0.98	≥6.7	95	95
**Shi et al., 2014 [23]**	To evaluate the ability of dual-energy CT (DECT) to differentiate adrenal adenomas from metastases using attenuation measurements at different energy levels.	Dual-source DECT (Siemens SOMATOM Definition Flash, Siemens Healthineers, Forchheim, Germany) with tube voltages of 80 kVp and 140 kVp, allowing for monoenergetic image reconstruction at 40–100 keV.	**Parameter**			**Adenomas (Mean ± SD, HU)**		**Metastases (Mean ± SD, HU)**		***p*-Value**
			**Mean Attenuation at 80 kVp**			10.1 ± 21.7		35.6 ± 8.4		<0.001
			**Mean Attenuation at 140 kVp**			18.4 ± 16.0		33.7 ± 7.6		<0.001
			**Mean Attenuation Change (MAVC 80–140 kVp, HU)**			8.2 ± 6.8		−1.8 ± 2.3		<0.001
			**Mean Attenuation at 40 keV**			−4.0 ± 32.7		38.7 ± 10.8		<0.001
			**Mean Attenuation at 100 keV**			18.9 ± 15.5		33.6 ± 7.6		<0.001
			**Mean Attenuation Change (MAVC 40–100 keV, HU)**			23.0 ± 19.3		−5.0 ± 6.2		<0.001
			**ROC Analysis for Differentiating Adenomas and Metastases**							
			**Parameter**	**AUC**	**Optimal Threshold**		**Sensitivity (%)**		**Specificity (%)**	
			**MAVC 80–140 kVp (HU)**	0.964	>2.42 HU		78.6		100	
			**MAVC 40–100 keV (HU)**	0.964	>6.95 HU		78.6		100	
**Winkelmann et al., 2022 [17]**	To evaluate the ability of dual-energy CT (DECT)-based iodine quantification, virtual non-contrast (VNC) imaging, and radiomic analysis in differentiating adrenal adenomas from metastases.	Dual-source CT (Siemens SOMATOM Definition Flash/Force, Siemens Healthineers, Forchheim, Germany) with postprocessing techniques including VNC imaging, iodine quantification, fat fraction analysis, and radiomics.	**Parameter**		**AUC**	**Optimal Threshold**		**Sensitivity (%)**	**Specificity (%)**	***p*-Value**
			**Virtual Non-Contrast (VNC) Attenuation (HU)**		0.89	≥13.07 HU		87.5	78.2	<0.001
			**Fat Fraction (%)**		0.86	≤17.20%		68.8	93.8	<0.001
			**Iodine Density (mg/mL)**		0.67	≤0.93 mg/mL		37.5	96.9	0.075
			**CT-Mixed Attenuation (HU)**		0.57	>61.33 HU		56.3	62.5	0.42
**Wu et al., 2023 [16]**	To assess the diagnostic value of spectral parameters in differentiating adrenal adenomas from metastases using dual-layer spectral CT (DLSCT).	Dual-layer spectral detector CT (Philips iQon Spectral CT, Philips Medical Systems, Cleveland, Ohio, United States).	**Parameter**	**Optimal Threshold**		**Sensitivity (%)**		**Specificity (%)**		**AUC**
			**Iodine-to-CTVNC Ratio**	≥4.18		74.4		91.9		0.920
			**CTVNC (HU)**	≤23		100.0		52.4		0.743
			**Slope of the Spectral HU Curve (s-SHC)**	≥2.1		95.4		42.9		0.723
			**Z-effective (Z-eff)**	≥8.25		93.0		42.9		0.706
			**Iodine Density (mg/mL)**	≥1.53		81.4		50.8		0.695
			**Normalized Iodine Density (NID)**	≥0.4		74.4		54.0		0.668

## Data Availability

The data presented in this study are available on request from the corresponding author.
